# Ruminant-Waste Protein Hydrolysates and Their Derivatives as a Bio-Flocculant for Oil Sands Tailing Management

**DOI:** 10.3390/polym13203533

**Published:** 2021-10-14

**Authors:** Jesse Yuzik, Vinay Khatri, Michael Chae, Paolo Mussone, David C. Bressler

**Affiliations:** 1 Department of Agricultural, Food and Nutritional Science, Faculty of Agricultural, Life and Environmental Sciences, University of Alberta, Edmonton, AB T6G 2P5, Canada; yuzik@ualberta.ca (J.Y.); vkhatri@ualberta.ca (V.K.); mchae@ualberta.ca (M.C.); 2 Applied BioNanotechnology Industrial Research Chair, Industry Solutions, Northern Alberta Institute of Technology, 10210 Princess Elizabeth Ave., NW, Edmonton, AB T5G 0Y2, Canada; PMUSSONE@nait.ca

**Keywords:** flocculant, crosslinking, peptides, glutaraldehyde, specified risk materials

## Abstract

Reclamation of tailings ponds is a critical issue for the oil industry. After years of consolidation, the slurry in tailings ponds, also known as fluid fine tailings, is mainly comprised of residual bitumen, water, and fine clay particles. To reclaim the lands that these ponds occupy, separation of the solid particles from the liquid phase is necessary to facilitate water removal and recycling. Traditionally, synthetic polymers have been used as flocculants to facilitate this process, but they can have negative environmental consequences. The use of biological polymers may provide a more environmentally friendly approach to flocculation, and eventual soil remediation, due to their natural biodegradability. Peptides derived from specified risk materials (SRM), a proteinaceous waste stream derived from the rendering industry, were investigated to assess their viability for this application. While these peptides could achieve >50% settling within 3 h in bench-scale settling tests using kaolinite tailings, crosslinking peptides with glutaraldehyde greatly improved their flocculation performance, leading to a >50% settling in only 10 min. Settling experiments using materials obtained through different reactant ratios during crosslinking identified a local optimum molar reactant ratio of 1:32 (peptide amino groups to glutaraldehyde aldehyde groups), resulting in 81.6% settling after 48 h. Taken together, these data highlight the novelty of crosslinking waste-derived peptides with glutaraldehyde to generate a value-added bioflocculant with potential for tailings ponds consolidation.

## 1. Introduction

In 2019, the Alberta oil sands generated 2.95 million barrels per day, making them the source of 63% of Canada’s oil production [1]. Isolation of a hydrocarbon-rich bitumen stream from oil sands is achieved through the Clarke hot water extraction method, in which hot water is injected into bitumen-rich layers of the ground to facilitate separation of bitumen and sediments [2]. The remaining byproduct, which consists of water with fine clays and some residual bitumen, is called tailings [2]. These tailings are stored indefinitely in tailings ponds, which are large, man-made ponds supported by dykes. With the inventory of these ponds growing by the day, it is essential to find economic and environmentally friendly ways to manage the tailings and reclaim the lands that they occupy.

Coagulation and flocculation are two basic strategies for consolidation and reclamation of tailings ponds [3]. Coagulants are multivalent cations (Ca^2+^, Al^3+^, and Fe^3+^) that reduce repulsion between solid particles by electrostatic, steric, and hydration forces, facilitating improved flocculation [4]. Flocculants are typically high molecular weight polymers that can bridge gaps between particles in a colloid to allow them to interact with each other and form larger particles (i.e., flocs), which can then settle out of solution [4,5]. Charged polymers are often used for the flocculation of the fine, negatively charged particles contained in tailings, forming strong ionic interactions that promote flocculation [4,6].

Currently, gypsum, a coagulant, and hydrolyzed polyacrylamide (HPAM), a flocculant, are widely used for the reclamation of tailings ponds. However, the synthetic polymer HPAM accumulates in the environment as it is not biodegradable and can also contain unreacted acrylamide, a known neurotoxin and potential carcinogen [7,8]. The development of bioflocculants can alleviate these concerns due to their improved biodegradability and sustainability. For example, chitosan is one of the most popular and highly studied bioflocculants due to its cationic nature [9,10]. Peptides, the building blocks of proteins, also have bioflocculant potential as charged groups inherent on the peptide backbone can interact with charged clay surfaces. However, due to their low molecular weight, peptides are often subjected to crosslinking, creating larger molecular weight polymers that are necessary to improve floc strength and formation rates, and thus induce rapid flocculation [11,12,13]. Glutaraldehyde is a well-known chemical crosslinker for peptides, with its two aldehyde groups known to react readily with amino groups of peptides at ambient conditions [14,15].

Specified risk materials (SRM) are protein-rich waste products from the beef rendering industry that can be transformed into high value products, including flocculants for wastewater treatment. SRM are certain tissues of livestock (i.e., brain, eyes, and spinal column) that have the potential to contain prion diseases, such as bovine spongiform encephalopathy (BSE), more commonly known as mad cow disease [16]. In 2007, the Canadian Food Inspection Agency banned the use of these materials in animal feeds, pet foods, and fertilizers, and mandated their disposal via land-filling or incineration [17]. However, this waste material can be rendered safe to use in other applications after it undergoes a thermal or alkaline hydrolysis, generating peptides in the process. Adding value to SRM-derived peptides would convert a costly and potentially dangerous waste with negative environmental impacts into a product that could ultimately provide an alternative revenue source for the rendering industry. Recent valorization approaches that have been examined by our laboratory include the conversion of SRM-derived peptides to plywood adhesives, torrefied wood pellet binders, tackifiers, and flocculants [12,18,19,20,21,22,23].

In this study, an SRM peptide–based flocculating agent was developed for tailings ponds treatment and recovery. Application of glutaraldehyde crosslinking to increase the molecular weight of these peptides was investigated and the resulting materials were subjected to bench-scale settling tests using synthetic kaolinite tailings. Finally, the use of different ratios of reagents during the crosslinking of peptides with glutaraldehyde was also assessed. Overall, this work provides insights into the development of a novel, biodegradable flocculant utilizing animal waste protein.

## 2. Materials

SRM were obtained from a large multinational rendering corporation in western Canada. The kaolinite clay used for bench-scale settling tests (i.e., synthetic kaolinite tailings) was Polygloss 90, which had a median particle size of 0.4 microns, and was obtained from KaMin LLC (Wrens, GA, USA). Calcium sulphate dihydrate (gypsum) (98+%) was obtained from Fisher Scientific (Fair Lawn, NJ, USA). Whatman no. 4 (pore size 20–25 mm) (GE Healthcare, Chicago, IL, USA) filter paper was obtained from Fisher Scientific and was used for all the vacuum filtration processes. A Buchi B290 Spray Dryer (BUCHI Corporation New Castle, DE, USA) was used to recover hydrolyzed peptides. The glutaraldehyde solution (50% Certified), methanol (HPLC grade, 99.9%), and concentrated HCl (Certified ACS Plus, 36.5 to 38.0%) were purchased from Fisher Scientific (Fair Lawn, NJ, USA).

### 2.1. Hydrolysis of SRM

SRM were hydrolyzed as per the protocol described by Mekonnen et al., 2015 [23]. In brief, hydrolysis took place by addition of distilled water to 1 kg of SRM in a 1:1 (wt/wt) ratio, followed by heating at 180 °C at ≥174 psi for 40 min in a 5.5 L reactor (Parr 4582, Parr Instrument Company, Moline, IL, USA) with constant stirring at 200 rpm, as per Canadian Food Inspection Agency regulations. After hydrolysis, the material is considered nonhazardous and was further processed to remove lipids and other insoluble material by centrifugation (Avanti J-26 XP high-performance centrifuge, Beckman Coulter Canada LP, Mississauga, ON, Canada) at 7000× *g* for 30 min, followed by a second round of centrifugation at 7000× *g* for 10 min. After each round of centrifugation, the liquid phase was decanted, and the solids were removed. Residual solids were further removed by vacuum filtration (Whatman No. 4 filter paper, Oakville, ON, Canada) of the liquid phase. After processing, the peptide mixture was spray-dried to remove water, with drying conditions consisting of an inlet temperature of 170 °C and an outlet temperature of 80 °C. The final product was a light brown powder.

### 2.2. Preparation of Synthetic Process Water

Synthetic process water (SPW) was used in our bench-scale settling tests to replicate the ion concentrations of a model oil sands tailings pond. It was prepared by adding the compounds to distilled water in the following concentrations: 0.055 g/L CaCl_2_·2H_2_O; 0.028 g/L KCl; 0.545 g/L NaCl; 0.443 g/L Na_2_SO_4_; 0.084 g/L MgCl_2_·6H_2_O; 0.895 g/L NaHCO_3_. Before the kaolinite slurry was prepared, the pH of the SPW was adjusted to 8.00 with concentrated HCl using a pH meter (Fisherbrand™ accumet™ AB15 Basic, Thermo Fisher Scientific, Ottawa, ON, Canada).

### 2.3. Preparation of Synthetic Kaolinite Tailings

To prepare the 4% (wt/wt) synthetic kaolinite tailings (i.e., kaolinite clay slurries), 10 g of kaolinite clay was added to 240 g of synthetic process water (SPW). When necessary, the SPW contained 300 ppm of gypsum (2H_2_O·CaSO_4_) as a coagulant. The slurries were then mixed on a jar tester (PB-900 jar tester from Phipps and Bird, Richmond, VA, USA) at 300 rpm for 30 min. Following this, the slurry was left to sit for 90 min to allow for full hydration of the clay particles. The mixture was then mixed again for 5 min immediately before use.

### 2.4. Bench-Scale Flocculation Experiments

**Figure 1:**
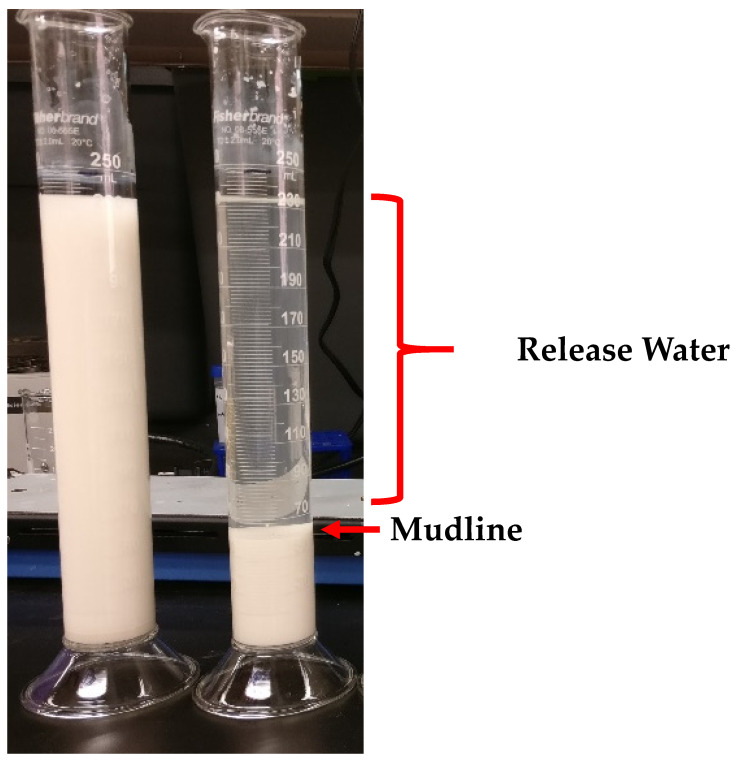
Example of a bench-scale flocculation experiment.

Bench-scale flocculation experiments were carried out in 250 mL graduated cylinders. A quantity of 250 g of synthetic kaolinite slurry, corresponding to 247 mL, was added to the graduated cylinders. Various treatments were added to the cylinders and the contents of the cylinders were then homogenized by plunging 20 times using a custom plunger, made by welding a metal rod to a washer. Flocculants were added on a wt/wt basis. Settling was recorded by measuring the height of the mudline over time. An example is shown in Figure 1.

### 2.5. Turbidity Measurements

The turbidity of the release water was measured using a turbidimeter (Orion AQ4500 Thermo Fisher Scientific, Grand Island, NY, USA) after 48 h of settling by withdrawing the supernatant with a pipette approximately halfway between the surface of the solution and the mudline. Samples were measured using the IR ratio mode to eliminate any interference from the color of the sample. The principle behind this measurement is that there is one light source and two detectors to measure the scattering of light. The light source sends a beam of light into the sample and is detected by detector 1, which is situated directly across from the light source, to record a reference measurement. At the same time, a second detector records the light that was scattered at a 90° angle to the light source, which is qualified as the active signal. An algorithm then determines the turbidity based on the ratio of these measurements and compensates for the color absorption in the reference beam. The range of the measurement method described by the manufacturer is from 0 to 4000 NTU (Nephelometric Turbidity Unit). The wavelength used for this measurement was 860 nm.

### 2.6. Crosslinking Reaction of Peptides with Glutaraldehyde

To increase the molecular weight of the peptides, they were reacted with the crosslinking agent glutaraldehyde. The peptides were dispersed in methanol, followed by a dropwise addition of the glutaraldehyde solution. This reaction was carried out at room temperature for 2 h with stirring. The product was recovered by vacuum filtration, followed by washing with methanol. Residual solvent was evaporated from the product in a fume hood at room temperature for 48 h. After drying, the resulting solid was ground to a powder with a mortar and pestle. The resulting product could be characterized as a fine brown colored powder.

**Figure 2:**
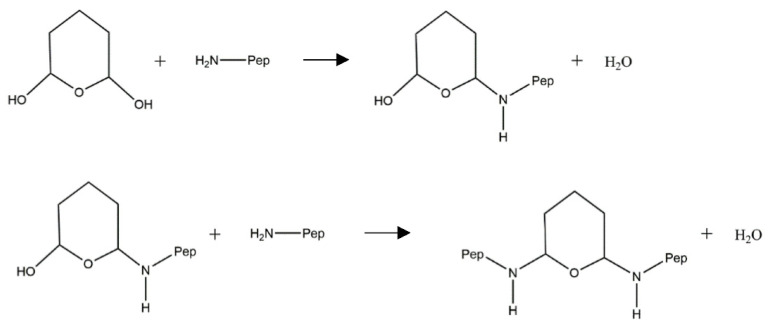
Crosslinking reaction scheme of glutaraldehyde with peptides. Glutaraldehyde can react with the amino groups of two peptide molecules to form a larger crosslinked structure. Adapted from Migneault et al. [24].

A reaction scheme for the glutaraldehyde crosslinking reaction is proposed in Figure 2. The glutaraldehyde can readily react with the amino group of the peptide to form the peptide–glutaraldehyde intermediate molecule. This reaction can occur at the other hydroxyl group on the peptide–glutaraldehyde intermediate resulting in the formation of a crosslinked peptide.

### 2.7. Varying the Crosslinking Ratio

The ratio of the reactants used during the crosslinking reaction was varied to determine the effect that altering the crosslinker amount would have on the products. An estimation of the amount of the amino and carboxylic acid groups of the SRM peptides was determined in a previous study to be 0.6 mmol/g for the amino groups and 1.6 mmol/g for the carboxylic acid groups [22]. The reactant ratio was varied by keeping the peptide amount constant and varying the amount of glutaraldehyde added based on the ratio of the aldehyde groups to the amino groups of the peptides. The reactant ratio was based on the reaction taking place solely with the amino groups, which was also shown in previous studies [14,15,24,25]. Therefore, the reactants were varied as shown in Table 1.

### 2.8. Thermal Gravimetric Analysis

Thermal gravimetric analysis (TGA) was used to determine the thermal stability of the peptide samples using a TGA Q50 from TA instruments (New Castle, DE, USA). The temperature was ramped to 100 °C with an increment of 10 °C/min, followed by an isothermal stage for 10 min, and finally the temperature was increased to 500 °C with an increment of 5 °C/min.

### 2.9. Size Exclusion High Performance Liquid Chromatography

Size exclusion chromatography–high performance liquid chromatography (SEC-HPLC) was done using a 1200 Series LC System from Agilent Technologies (Mississauga, ON, USA) with a diode array detector to measure in the UV range at 210 nm, based on a method from previous research [11]. Superdex Peptide 10/300 GL and Superdex 200 Increase 10/300 GL columns (GE Healthcare Biosciences AB, Uppsala, Sweden) were used in series to enhance separation of the molecules. The Superdex Peptide column has a separation range from 100–7000 Da and the Superdex 200 column has a range of 10–600 kDa. The mobile phase used was a 0.15 M Na_2_HPO_4_ solution adjusted to pH 9 with NaOH. Sample injection was done at 0.5 mL/min with an injection volume of 20 µL. A series of standards of blue dextran (2000 kDa), alcohol dehydrogenase (150 kDa), albumin (66 kDa), carbonic anhydrase (29 kDa), cytochrome C (12.4 kDa), aprotinin (6.5 kDa), and Vitamin B-12 (1.36 kDa) were used to standardize the retention times of the products and were obtained from Sigma-Aldrich (St. Louis, MO, USA).

### 2.10. Sodium Dodecyl Sulfate-Polyacrylamide Gel Electrophoresis

The method used for gel electrophoresis was a tricine-SDS PAGE approach based on a protocol by Schägger and was run on a mini-protean II electrophoresis unit (Bio-Rad Laboratories, Richmond, CA, USA) [26]. Modifications were made to the protocol to obtain better results with the crosslinked peptides. To improve the solubility of the crosslinked peptides and loading into the gel, the samples were incubated in the 1X sample buffer solution and mixed for 2 h on a nutating mixer prior to gel loading. A quantity of 20 µL of the samples were loaded into the gel. The concentrations of the peptide sample solutions were optimized to allow proper visualization. The 0.5% and 0.1% (wt/wt) solutions were prepared for the peptides and a 10X more dilute solution was required for the crosslinked peptides at 0.05% and 0.01% (wt/wt). To obtain the lower concentration samples, dilution with the 1X sample buffer solution was performed. Once loaded, the samples were run at 100 V until they left the loading gel and were run at 200 V until the separation was complete. The gel was immediately transferred to a container to be visualized by a silver staining kit. The silver staining kit was a Pierce Silver Staining Kit obtained from Thermo Scientific and the method used was defined by the manufacturer [27]. After staining, the gels were imaged on an AlphaImager HP gel visualizing system from Alpha Innotech (San Leandro, CA, USA).

### 2.11. Fourier-Transform Infrared Spectroscopy

Assessment of the functional groups present within the peptides and the glutaraldehyde-crosslinked peptides was achieved using Fourier-Transform infrared spectroscopy (FTIR). Analysis was performed using an Attenuated Total Reflectance (ATR) model ALPHA from Bruker Optik GmbH (Rudolf-Plank-StraBe 27, 76275 Ettlingen, Germany). ATR was used as it provides a fast and convenient way to analyze solid samples.

### 2.12. Statistical Analyses

Statistical analyses were performed using Prism 9.1.2. (San Diego, CA, USA). Data were analyzed using one-way analysis of variance (ANOVA), with a Tukey HSD test applied post hoc at a 95% confidence level. To facilitate statistical analyses, all experiments were performed in triplicate unless otherwise indicated.

## 3. Results and Discussion

### 3.1. Glutaraldehyde Crosslinking Reaction with Peptides

Previous work had established that SRM-derived peptides could improve flocculation of a synthetic kaolinite slurry, albeit at relatively slow rates [12]. To improve flocculation, a glutaraldehyde crosslinking reaction was investigated, with the goal to increase the molecular weight of the peptides since larger molecules have been shown to have better flocculation characteristics [5,13,28]. The crosslinking chemical glutaraldehyde was investigated due to its relatively low cost and high reactivity. When attempting this reaction in an aqueous environment, the recovered products were tightly bound to the filter paper, resulting in a low recovery and contamination with filter paper. Therefore, different solvents were then investigated with methanol eventually chosen due to its high miscibility in water and its polar protic nature. Methanol has a pKa of 15.5, which is similar to the pKa of water at 15.7. In addition, the peptides were less soluble in methanol compared to water, due to its lower polarity, which allowed for better product recovery. The SRM-derived peptides were reacted with glutaraldehyde at room temperature for two hours in methanol at a molar amine–aldehyde ratio of 1:8 and others as described in Table 1.

### 3.2. Characterization of the Glutaraldehyde-Crosslinked Peptides

The products generated from crosslinking of peptides with glutaraldehyde were characterized in order to identify any changes to the structure of the peptides that occurred after the reaction. Theoretically, the crosslinking reaction should have occurred at the amino groups of the peptides to create more thermally stable, higher molecular weight products (Figure 2). Several analytical techniques were used to characterize the peptide–glutaraldehyde-crosslinked products including TGA, SDS-PAGE, and SEC-HPLC. It should also be noted that ATR-FTIR was also performed (Supplemental Figure S1), but since crosslinking of peptides with glutaraldehyde does not result in the formation of new types of chemical bonds, these data cannot be used to assess the occurrence of crosslinking.

### 3.3. TGA of Glutaraldehyde-Crosslinked Peptides

**Figure 3:**
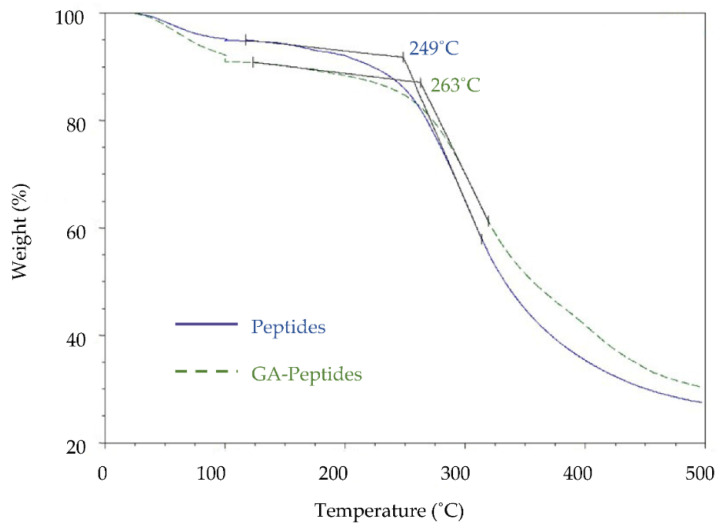
TGA data acquired using peptides (Peptides) or glutaraldehyde-crosslinked peptides (GA-Peptides; 1:8 ratio). The onset of the thermal degradation was determined using the ASTM standard method E2550-17.

The first analysis that was used to characterize the glutaraldehyde-crosslinked peptides was TGA (Figure 3). The onset of degradation can be determined by finding the intercept of two extrapolated lines, one from the flat base before decomposition, and the other from the sharpest slope, as described by ASTM standard method E2550–17. For the unmodified peptides, the onset of degradation temperature was 249 °C, while for the glutaraldehyde-crosslinked peptides it was 263 °C. This shift signifies that the glutaraldehyde-crosslinked peptides were more thermally stable and required a higher temperature to break the additional bonds formed during the reaction with glutaraldehyde. Another interesting note is that at 500 °C the unmodified peptides had a lower weight % remaining than the crosslinked peptides, indicating that the material had increased thermal stability at higher temperatures, again likely due to the greater number of chemical bonds in the crosslinked product. The increased bonding suggests that higher molecular weight compounds were formed during the crosslinking of peptides with glutaraldehyde.

### 3.4. SEC-HPLC Analysis of Glutaraldehyde-Crosslinked Peptides

**Figure 4:**
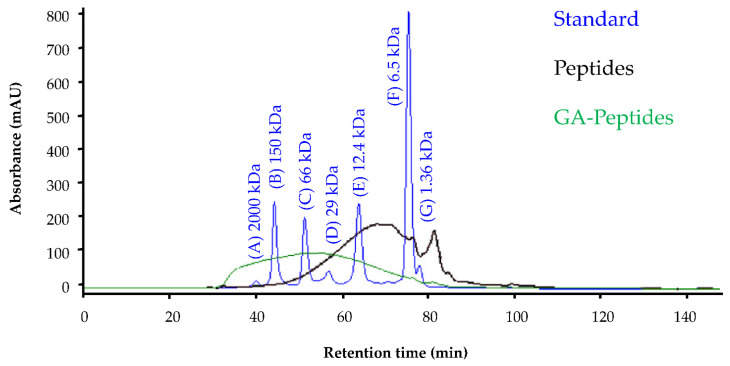
Results from the SEC-HPLC analysis of the peptides (Peptides) and the soluble portion of the glutaraldehyde-crosslinked peptides (GA-Peptides; 1:8 ratio). A 0.15 M Na_2_HPO_4_ solution was used as the mobile phase using HPLC grade reagents. Standards used to assess the retention times of the products were (A) blue dextran (2000 kDa), (B) alcohol dehydrogenase (150 kDa), (C) albumin (66 kDa), (D) carbonic anhydrase (29 kDa), (E) cytochrome c (12.4kDa), (F) aprotinin (6.5 kDa), and (G) Vitamin B-12 (1.36 kDa).

To confirm the changes in the molecular weight of the peptides after the glutaraldehyde crosslinking reaction, SEC-HPLC was attempted. However, the material recovered after the glutaraldehyde crosslinking reaction was much less soluble. While this physical change further supports the occurrence of crosslinking, it is also problematic because SEC-HPLC requires that the analyte is completely soluble in the mobile phase. Nevertheless, the soluble material present after crosslinking was analyzed (Figure 4). From these data, the soluble portion of the glutaraldehyde-crosslinked peptides are observed to be distributed in a higher molecular weight region than the unmodified peptides. This further suggests that the crosslinking reaction of peptides with glutaraldehyde was successful.

### 3.5. SDS-PAGE Analysis of Glutaraldehyde-Crosslinked Peptides

To further confirm the crosslinking of the SRM-derived peptides with glutaraldehyde, a commonly used molecular biology technique, sodium dodecyl sulfate–polyacrylamide gel electrophoresis (SDS-PAGE), was also performed. One of the advantages of SDS-PAGE over SEC-HPLC is that it uses a surfactant, sodium dodecyl sulphate (SDS), which is normally added to help denature the protein structure. It was found that the SDS was also able to improve the solubility of the crosslinked peptides in solution.

**Figure 5:**
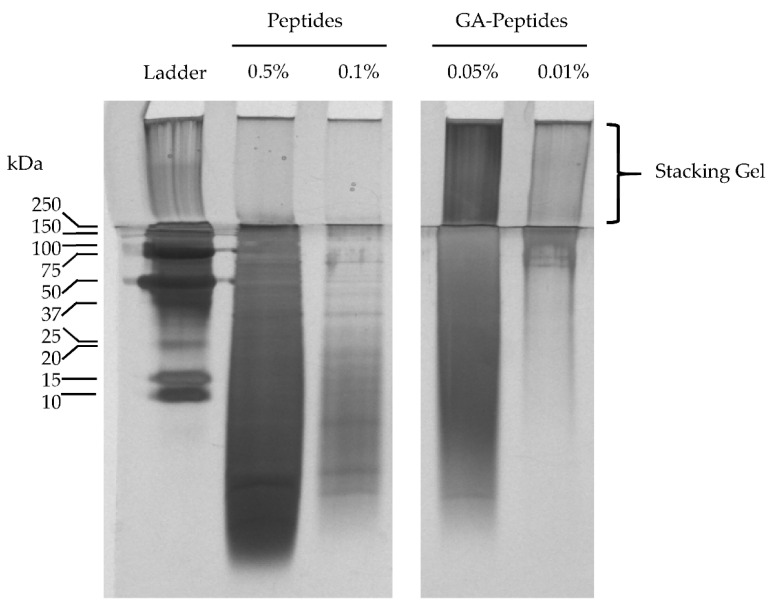
SDS-PAGE of unmodified peptides and glutaraldehyde-crosslinked peptides (1:8 ratio) after silver staining. The samples include unmodified peptides (Peptides) and the glutaraldehyde-crosslinked peptides (GA-Peptides). The 0.5% and 0.05% solutions were prepared by adding 5 mg and 0.5 mg, respectively, of sample to 1 mL of 1X sample buffer. The 0.1% and 0.01% solutions were prepared through 1/5 dilutions of the 0.5% and 0.05% solutions, respectively. Samples were dissolved in sample buffer for 2 h prior to loading of 20 µL in each lane. It should be noted that all lanes shown were from the same gel, but the middles lanes were removed as they contained samples that were not relevant to this study.

A silver staining procedure was used to stain the gel after SDS-PAGE was performed to visualize the samples. As shown in the gel in Figure 5, two loading amounts of both the peptides and glutaraldehyde-crosslinked peptides were analyzed to obtain a better visualization of the products in different molecular weight ranges. As shown in the lane containing 0.01% glutaraldehyde-crosslinked peptide (GA-Peptides; 0.01%), most of the material observed was in the ≥75 kDa range, which was much higher than the range observed in the 0.1% peptide treatment lane (Peptides: 0.1% Pep). This further confirmed that the glutaraldehyde-crosslinked peptides had a much higher molecular weight than the unmodified peptides, providing additional evidence that the crosslinking reaction with glutaraldehyde was successful.

Also of note, there is a visible amount of material in the crosslinked peptide samples found in the stacking gel, which likely has a molecular weight that is ≥250 kDa. This material may be part of the insoluble material that was not able to dissolve in the HPLC mobile phase and was therefore not analyzed. This would explain why there was a difference in the range of the molecular weights from the SDS-PAGE and the SEC-HPLC, and thus demonstrates the advantage to using SDS-PAGE when analyzing the glutaraldehyde-crosslinked peptides.

### 3.6. Flocculation of a Kaolinite Clay Slurry with Glutaraldehyde-Crosslinked Peptides

**Figure 6:**
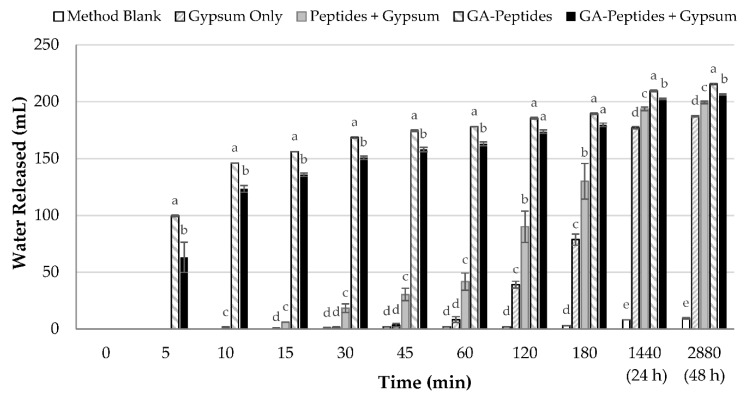
Flocculation using glutaraldehyde-crosslinked peptides (1:8 ratio). A 4% (wt/wt) kaolinite slurry was used. Gypsum was added as a coagulant when necessary, at a concentration of 300 ppm. Flocculants were added at a 3% dosage (wt/wt), and the difference between the mudline and the initial height of the slurry was recorded as the water released. As controls, the kaolinite slurry was subjected to settling tests after no flocculant addition (Method Blank) or with the addition of gypsum alone (Gypsum Only). Unmodified peptides were also used as a flocculant with gypsum (Peptides + Gypsum). For the peptides that were crosslinked with glutaraldehyde, settling experiments were performed in both the absence (GA- Peptides) and presence (GA-Peptides + Gypsum) of gypsum. Experiments were performed in triplicate, except for GA-Peptides, which was performed in duplicate. Within each time point, columns with different letters (a-e) above them are significantly different at a 95% confidence level.

The crosslinked products that were characterized above were then tested in flocculation experiments. The main objective of this experiment was to determine whether application of the glutaraldehyde-crosslinked peptides to a model kaolinite clay slurry would result in a faster settling rate than the unmodified peptides. As shown in Figure 6 and Supplemental Table S1, after only five minutes, the glutaraldehyde-crosslinked peptides led to a significantly higher amount of settling, with and without gypsum, than the unmodified peptides and the other treatments. In fact, it took 120 min of settling before the unmodified peptides reached a similar settling volume to that achieved after 5 min using the glutaraldehyde-crosslinked peptides. These data clearly demonstrate that crosslinking SRM-derived peptides with glutaraldehyde dramatically improved their settling performance. Even after 48 h, the settling achieved using the glutaraldehyde-crosslinked peptides (either with or without gypsum) was significantly higher than that achieved using the unmodified peptides. Crosslinking of peptides with glutaraldehyde leads to an increase in molecular weight, which results in improved bridging of suspended solid particles. Improved flocculation resulting from an increase in molecular weight of the flocculant has been observed in many other systems described in the literature [12,13,29].

**Figure 7:**
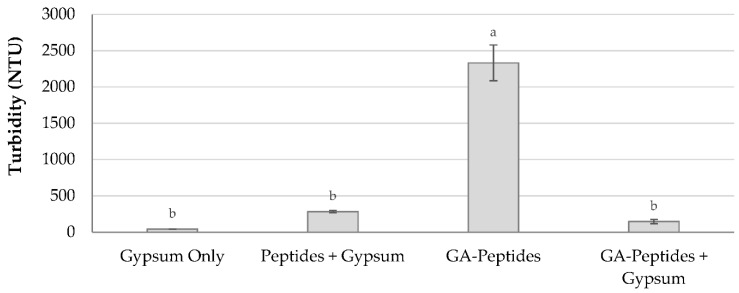
Turbidity after flocculation with glutaraldehyde-crosslinked peptides (1:8 ratio). The turbidity averages of the different treatments after 48 h of settling in a 4% (wt/wt) kaolinite clay system. Gypsum was added as a coagulant at a concentration of 300 ppm. Experiments were performed in triplicate, except for GA-Peptides, which was performed in duplicate. Columns with different letters (a-b) above them have significantly different turbidity at a 95% confidence level.

Interestingly, the settling data showed that the peptides crosslinked with glutaraldehyde did not require gypsum as a coagulant to settle the slurry, as is the case with the incumbent industrial flocculant, HPAM. In fact, the peptides crosslinked with glutaraldehyde had a faster settling rate when gypsum was excluded from the settling cylinder (Figure 6). However, when comparing the turbidity data after 48 h of settling, the treatment without gypsum had a much higher turbidity than the treatment with gypsum (Figure 7). This indicates that there is a trade-off between a slightly faster settling rate and a lower final turbidity, when gypsum is absent or present. This trade-off has also been shown in other flocculation studies. Wang et al. (2010) looked at two versions of polyacrylamide (PAM), one cationic and one anionic, for tailings settling [30]. They found that the PAM with the higher initial settling rate (43 m/h) had a higher turbidity (470 NTU) compared to the other PAM, which had slower initial settling rate (20 m/h), but a lower turbidity (68 NTU). When the turbidity of the release water is higher it indicates that there are some particles remaining in solution and, thus, when gypsum is present, fewer particles are remaining in the solution. Ultimately, the use of gypsum will depend on the application, and whether the settling rate or turbidity is the more crucial factor.

### 3.7. Varying Reactant Ratio during Peptide Crosslinking

Next, the ratio of the two functional groups present in the reactants, glutaraldehyde and peptides, was differed and the effect on settling performance was studied. In these experiments, the amount of the peptide amino groups was kept consistent and the amount of glutaraldehyde was varied. It should be noted that due to the large number of measurements required to assess flocculation performance in graduated cylinders, particularly within the first 60 min of the experiment, only two formulations (and associated controls, all performed in triplicate) could be tested in a single experiment. Thus, the 1:2 and 1:4 formulations were tested first, followed by the 1:18 and 1:16 formulations, and finally the 1:32 and 1:64 formulations.

**Figure 8:**
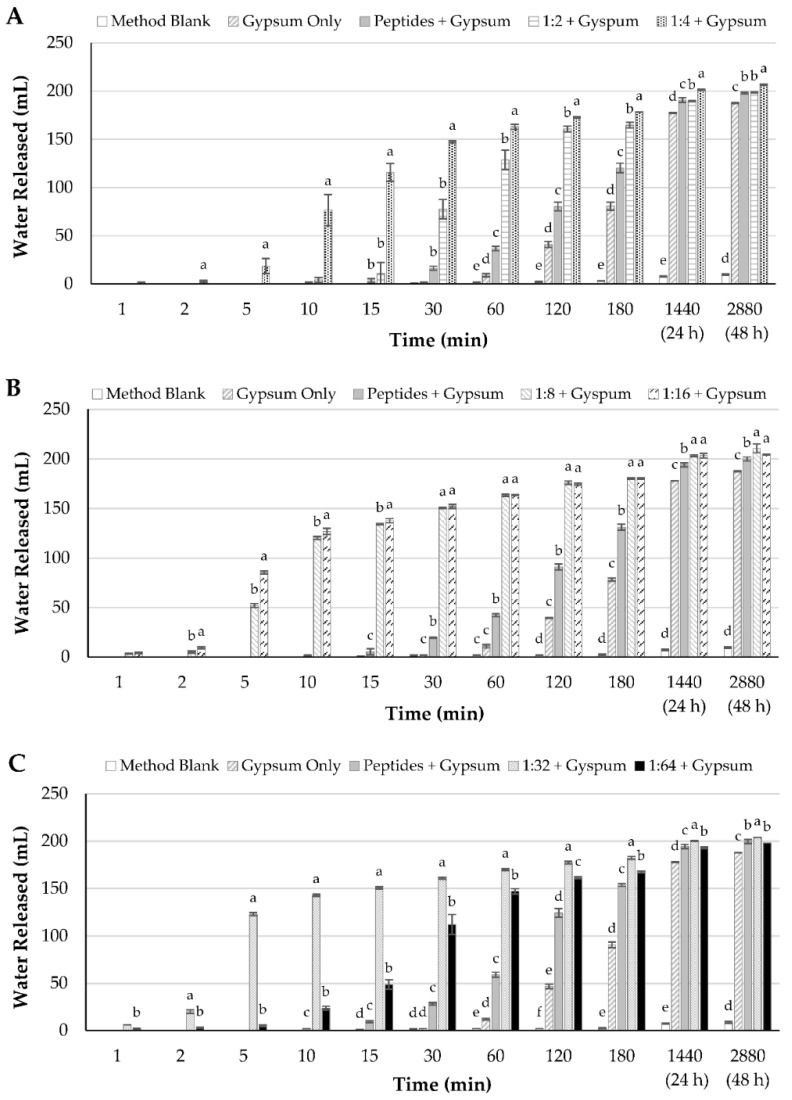
Flocculation performance of glutaraldehyde-crosslinked peptides generated using different ratios of reactant functional groups (amino groups in peptides–aldehyde groups in glutaraldehyde): 1:2 and 1:4 (**A**); 1:8 and 1:16 (**B**); and 1:32 and 1:64 (**C**). Flocculation of a 4% (wt/wt) kaolinite slurry was observed over a 48-h time period. For the method blank, no additional materials were added. Where indicated, gypsum was added as a coagulant at a concentration of 300 ppm. The various flocculants were added at 3% (wt/wt). Unmodified peptides (Peptides) were used as a baseline to assess the impacted of crosslinking. The difference between the mudline and the initial height of the slurry was recorded as the water released. Experiments were performed in triplicate. Within each time point, columns with different letters (a–e) above them are significantly different at a 95% confidence level.

As shown in Figure 8A and Supplemental Table S2, the product generated from a 1:4 ratio outperformed that produced using a 1:2 ratio, both in terms of the settling rate and the final water release after 48 h. Conversely, the 1:16 ratio flocculant resulted in a higher amount of settling compared to the 1:8 ratio material for the first 10 min of settling (Figure 8B and Supplemental Table S3). However, after this point there was no longer a statistically significant difference between the groups until 48 h of settling, where the product from the 1:8 ratio induced slightly more settling than that from the 1:16 ratio. Finally, the material generated from a 1:32 ratio outperformed that from the 1:64 ratio with regards to settling rate and final water release (Figure 8C and Supplemental Table S4).

Although the results from the three panels cannot be directly compared, there was a general trend suggesting that increasing the reactants ratio higher than 1:2 results in a product with better flocculation properties; conversely, decreasing the ratio from 1:32 to 1:64 led to a product with reduced flocculation performance. Taken together, these data suggest that the optimal reactant ratio was between 1:2 and 1:64. Furthermore, looking at the data obtained after 5 min of settling, the general trend revealed that as the reactant ratio was increased from 1:2 to 1:32, there was substantial improvement in the settling performance of the crosslinked peptide before falling off again when the reactant ratio was decreased to 1:64. This suggests that a local optimum in settling performance was reached with the 1:32 reactants ratio. It should be noted that other variables during crosslinking and settling experiments (i.e., pH, temperature, etc.) could impact flocculation performance and floc morphology. These variables will be studied in future experiments.

**Figure 9:**
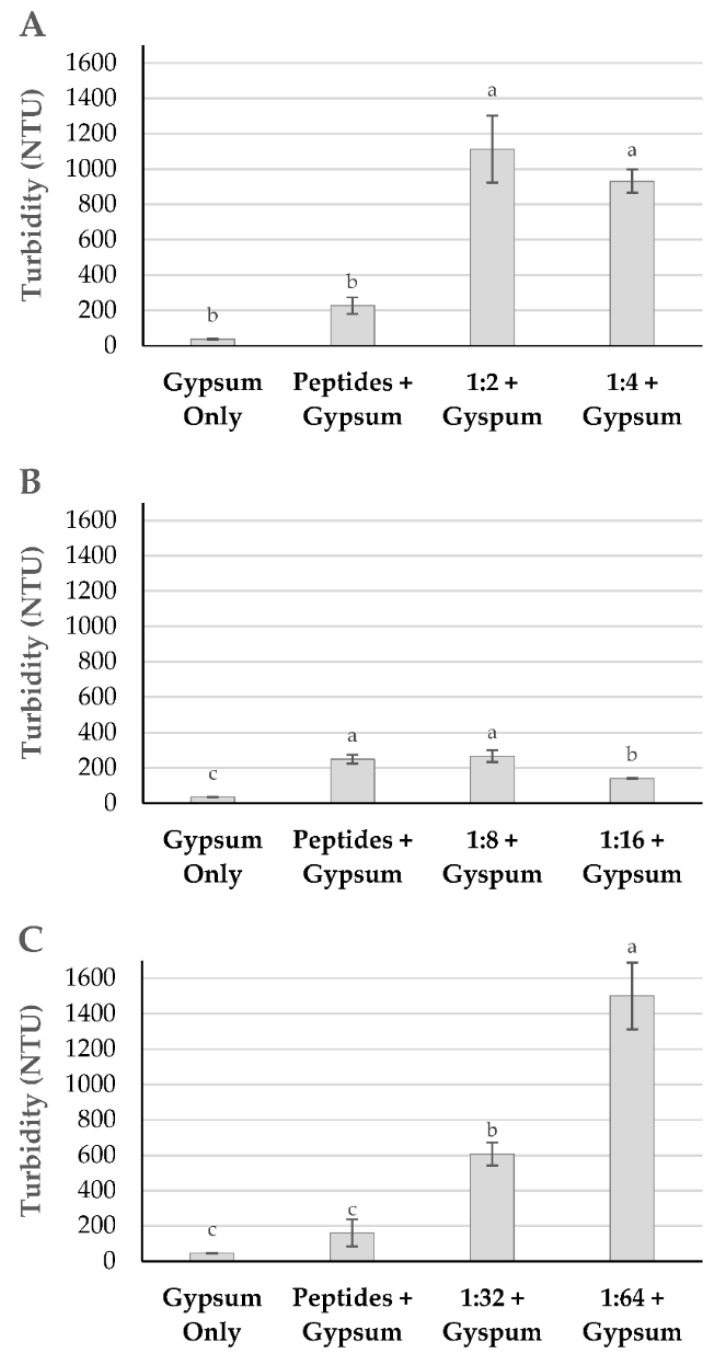
Turbidity of release water after flocculation using unmodified peptides (Peptides) or glutaraldehyde-crosslinked peptides generated using different ratios of reactant functional groups (amino groups in peptides–aldehyde groups in glutaraldehyde): 1:2 and 1:4 (**A**); 1:8 and 1:16 (**B**); and 1:32 and 1:64 (**C**). In flocculation experiments, gypsum was added as a coagulant at a concentration of 300 ppm. Turbidity data are reported as averages of triplicates after 48 h of settling in a 4% (wt/wt) kaolinite clay system. Within each panel, columns with different letters (a–c) above them have significantly different turbidity at a 95% confidence level.

The turbidity of the released water was also measured after 48 h of settling with the flocculants generated using different molar ratios of reactants. As shown in Figure 9A, there was no statistical difference in turbidity observed between the flocculants generated at a 1:2 and 1:4 reactants ratio. A large improvement in turbidity was attained when the ratio was reduced to 1:8 and 1:16, with the latter ratio generating a product with a slightly lower turbidity. Further decreases in the reactant ratio to 1:32 and 1:64 led to successive increases in turbidity, with the 1:64 ratio resulting in the highest turbidity of all ratios tested. Taken together, these data demonstrate that the flocculant derived using a reactants ratio of 1:16 displayed the best water clarity, performing slightly better than that generated from a reactants ratio of 1:8.

## 4. Conclusions

This research provides a proof-of-concept that waste materials from the beef rendering industry could be valorized through conversion into an effective bioflocculant for tailings ponds applications. Peptides were successfully crosslinked with glutaraldehyde leading to molecules with substantially higher molecular weight, as determined through liquid chromatography, thermogravimetric analysis, and gel electrophoresis. The settling rates achieved using the crosslinked peptides were significantly enhanced relative to what was observed using the unmodified peptides, particularly within the first hour of the experiment. Furthermore, the release water generated using the crosslinked peptides was significantly less turbid than that obtained using unmodified peptides. Altering the ratio of amino groups in the peptides and aldehyde groups in glutaraldehyde during crosslinking had a significant impact on the settling performance of the resulting flocculant, and also impacted the turbidity of the release water. Thus, selection of the appropriate amino to aldehyde ratio depends on whether the settling rate, final water release, or final turbidity is of greatest importance for a particular application. Taken together, these data demonstrate the great potential of using glutaraldehyde-crosslinked peptides as a bioflocculant for tailings management.

## Figures and Tables

**Table 1:** Variation of the molar ratios of the reactants in the glutaraldehyde–peptide crosslinking reactions. The ratios were based on the estimated amino groups of the peptides to the aldehyde functional groups of glutaraldehyde. Reactions were performed in triplicate.

Reactant Ratio	Peptides (g)	Glutaraldehyde (50% Solution) (mL)	Methanol (mL)
1:2	4.00	0.426	100
1:4	4.00	0.852	100
1:8	4.00	1.70	100
1:16	4.00	3.41	100
1:32	4.00	6.82	100
1:64	4.00	13.6	100

## Data Availability

Not applicable.

## Supplementary Materials

The following are available online at https://www.mdpi.com/article/10.3390/polym13203533/s1.
