# Supplementary material for: Ruminant-Waste Protein Hydrolysates and Their Derivatives as a Bio-Flocculant for Oil Sands Tailing Management
Source: Polymers (Basel). 2021 Oct 14;13(20):3533. doi: 10.3390/polym13203533 (PMC8538817; doi:10.3390/polym13203533)
Supplement: Supplementary file 1 [file polymers-13-03533-s001.zip › polymers-1381615-supplementary.pdf]

polymers

MDPI

Supplemental Materials:

Ruminant-Waste Protein Hydrolysates and Their Derivatives as a Bio-Flocculant for Oil Sands Tailing Management

Jesse Yuzik [1], Vinay Khatri [1], Michael Chae [1], Paolo Mussone [2] and David C. Bressler [1,*]

[1] Department of Agricultural, Food and Nutritional Science, Faculty of Agricultural, Life and Environmental Sciences, University of Alberta, Edmonton, AB T6G 2P5, Canada; yuzik@ualberta.ca (J.Y.); vkhatri@ualberta.ca (V.K.); mchae@ualberta.ca (M.C.)

[2] Applied BioNanotechnology Industrial Research Chair, Industry Solutions, Northern Alberta Institute of Technology, 10210 Princess Elizabeth Ave., NW, Edmonton, AB T5G 0Y2, Canada; PMUSSONE@nait.ca

* Correspondence: dbressle@ualberta.ca

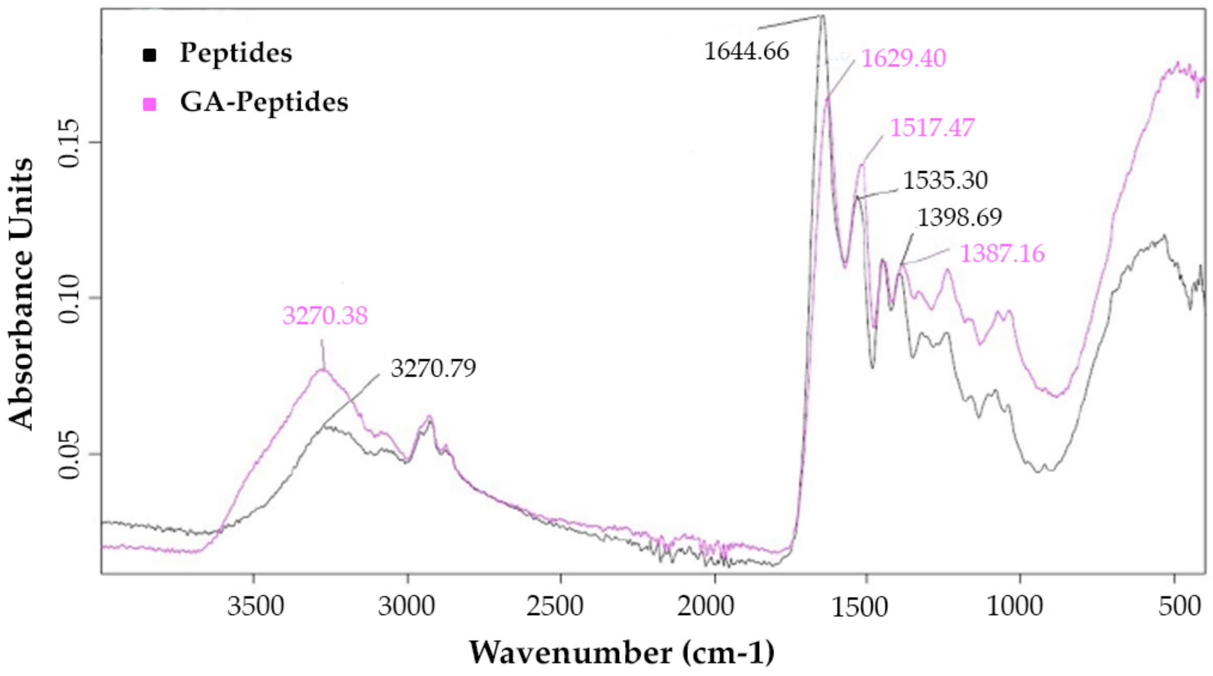

Figure S1. Attenuated Total Reflectance (ATR) Fourier-Transform Infrared Spectroscopy (FTIR) spectra of SRM-derived peptides (Peptides) and peptides crosslinked with glutaraldehyde (GA-Peptides; 1:8 ratio).

Supplemental Table S1. Percent settling over time in experiments designed to assess the impact of glutaraldehyde cross-linking on the flocculation performance of SRM-derived peptides.

	**Method Blank**	**Gypsum Only**	**Peptides + Gypsum**	**GA-Peptides***	**GA-Peptides* + Gypsum**
**0 min**	0.00 ± 0.00%	0.00 ± 0.00%	0.00 ± 0.00%	0.00 ± 0.00%	0.00 ± 0.00%
**5 min**	0.00 ± 0.00%	0.00 ± 0.00%	0.00 ± 0.00%	39.8 ± 0.3%	25.2 ± 5.3%
**10 min**	0.00 ± 0.00%	0.00 ± 0.00%	0.53 ± 0.23%	58.4 ± 0.0%	49.3 ± 1.2%
**15 min**	0.00 ± 0.00%	0.13 ± 0.23%	2.40 ± 0.00%	62.4 ± 0.0%	54.3 ± 0.6%
**30 min**	0.27 ± 0.23%	0.53 ± 0.23%	7.33 ± 1.51%	67.4 ± 0.3%	60.3 ± 0.6%
**45 min**	0.80 ± 0.00%	1.33 ± 0.61%	12.1 ± 2.2%	69.8 ± 0.3%	63.2 ± 0.8%
**60 min**	0.80 ± 0.00%	3.33 ± 1.01%	16.7 ± 3.0%	71.2 ± 0.0%	65.2 ± 0.7%
**120 min**	0.80 ± 0.00%	15.6 ± 1.2%	36.0 ± 5.5%	74.2 ± 0.3%	69.5 ± 0.6%
**180 min**	1.20 ± 0.00%	31.5 ± 2.0%	52.0 ± 6.2%	75.8 ± 0.3%	71.9 ± 0.6%
**24 h**	3.20 ± 0.00%	70.8 ± 0.4%	77.5 ± 0.6%	83.8 ± 0.3%	80.9 ± 0.2%
**48 h**	3.73 ± 0.23%	74.9 ± 0.2%	79.7 ± 0.5%	86.2 ± 0.3%	82.5 ± 0.2%

* Glutaraldehyde crosslinked peptides were obtained using a 1:8 molar ratio (amino:aldehyde groups).

Supplemental Table S2. Percent settling over time in experiments designed to assess the flocculation capacity of bioflocculants generated using crosslinking ratios of 1:2 and 1:4 (amino:aldehyde groups).

	Method Blank	Gypsum Only	Peptides + Gypsum	1:2 + Gypsum	1:4 + Gypsum
**0 min**	0.00 ± 0.00%	0.00 ± 0.00%	0.00 ± 0.00%	0.00 ± 0.00%	0.00 ± 0.00%
**1 min**	0.00 ± 0.00%	0.00 ± 0.00%	0.00 ± 0.00%	0.00 ± 0.00%	0.80 ± 0.00%
**2 min**	0.00 ± 0.00%	0.00 ± 0.00%	0.00 ± 0.00%	0.00 ± 0.00%	1.20 ± 0.40%
**5 min**	0.00 ± 0.00%	0.00 ± 0.00%	0.00 ± 0.00%	0.00 ± 0.00%	7.47 ± 3.11%
**10 min**	0.00 ± 0.00%	0.00 ± 0.00%	0.80 ± 0.00%	1.73 ± 1.01%	30.7 ± 6.4%
**15 min**	0.00 ± 0.00%	0.00 ± 0.00%	1.33 ± 0.92%	4.27 ± 4.69%	46.3 ± 3.7%
**30 min**	0.40 ± 0.00%	0.80 ± 0.00%	6.53 ± 0.83%	31.1 ± 4.0%	58.9 ± 0.5%
**60 min**	0.80 ± 0.00%	3.60 ± 0.69%	14.7 ± 0.9%	51.5 ± 4.0%	65.2 ± 1.1%
**120 min**	0.93 ± 0.23%	16.4 ± 1.2%	32.1 ± 1.8%	64.3 ± 1.2%	69.1 ± 0.2%
**180 min**	1.20 ± 0.00%	32.3 ± 1.7%	48.1 ± 2.0%	65.9 ± 1.2%	71.2 ± 0.0%
**24 h**	3.07 ± 0.23%	70.9 ± 0.2%	76.3 ± 0.9%	75.9 ± 0.2%	80.5 ± 0.2%
**48 h**	3.87 ± 0.23%	75.1 ± 0.2%	79.2 ± 0.4%	79.5 ± 0.2%	82.7 ± 0.2%

Supplemental Table S3. Percent settling over time in experiments designed to assess the flocculation capacity of bioflocculants generated using crosslinking ratios of 1:8 and 1:16 (amino:aldehyde groups).

	Method Blank	Gypsum Only	Peptides + Gypsum	1:8 + Gypsum	1:16 + Gypsum
**0 min**	0.00 ± 0.00%	0.00 ± 0.00%	0.00 ± 0.00%	0.00 ± 0.00%	0.00 ± 0.00%
**1 min**	0.00 ± 0.00%	0.00 ± 0.00%	0.00 ± 0.00%	1.33 ± 0.23%	1.73 ± 0.23%
**2 min**	0.00 ± 0.00%	0.00 ± 0.00%	0.00 ± 0.00%	2.00 ± 0.40%	3.73 ± 0.46%
**5 min**	0.00 ± 0.00%	0.00 ± 0.00%	0.00 ± 0.00%	20.8 ± 0.8%	34.3 ± 0.6%
**10 min**	0.00 ± 0.00%	0.00 ± 0.00%	0.53 ± 0.23%	48.1 ± 0.6%	50.8 ± 1.2%
**15 min**	0.00 ± 0.00%	0.13 ± 0.23%	2.13 ± 1.22%	53.7 ± 0.2%	55.1 ± 0.8%
**30 min**	0.53 ± 0.23%	0.80 ± 0.00%	7.87 ± 0.23%	60.3 ± 0.2%	60.8 ± 0.8%
**60 min**	0.80 ± 0.00%	4.40 ± 0.69%	16.9 ± 0.6%	65.3 ± 0.5%	65.5 ± 0.2%
**120 min**	0.80 ± 0.00%	15.9 ± 0.2%	36.4 ± 1.2%	70.4 ± 0.7%	69.9 ± 0.5%
**180 min**	0.93 ± 0.23%	31.3 ± 0.6%	52.5 ± 1.2%	72.1 ± 0.2%	72.1 ± 0.2%
**24 h**	2.93 ± 0.23%	71.2 ± 0.0%	77.6 ± 0.8%	81.3 ± 0.5%	81.3 ± 0.9%
**48 h**	3.87 ± 0.23%	75.1 ± 0.2%	80.0 ± 0.8%	84.3 ± 1.8%	81.7 ± 0.2%

Supplemental Table S4. Percent settling over time in experiments designed to assess the flocculation capacity of bioflocculants generated using crosslinking ratios of 1:32 and 1:64 (amino:aldehyde groups).

	Method Blank	Gypsum Only	Peptides + Gypsum	1:32 + Gypsum	1:64 + Gypsum
**0 min**	0.00 ± 0.00%	0.00 ± 0.00%	0.00 ± 0.00%	0.00 ± 0.00%	0.00 ± 0.00%
**1 min**	0.00 ± 0.00%	0.00 ± 0.00%	0.00 ± 0.00%	2.40 ± 0.00%	0.67 ± 0.23%
**2 min**	0.00 ± 0.00%	0.00 ± 0.00%	0.00 ± 0.00%	8.00 ± 0.80%	1.07 ± 0.23%
**5 min**	0.00 ± 0.00%	0.00 ± 0.00%	0.00 ± 0.00%	49.2 ± 0.7%	2.00 ± 0.40%
**10 min**	0.00 ± 0.00%	0.00 ± 0.00%	0.80 ± 0.00%	57.2 ± 0.4%	9.47 ± 0.83%
**15 min**	0.00 ± 0.00%	0.27 ± 0.23%	3.73 ± 0.46%	60.3 ± 0.5%	19.5 ± 2.0%
**30 min**	0.53 ± 0.23%	0.80 ± 0.00%	11.3 ± 0.6%	64.3 ± 0.5%	44.8 ± 4.2%
**60 min**	0.80 ± 0.00%	4.80 ± 0.40%	23.6 ± 1.1%	68.0 ± 0.4%	58.8 ± 1.1%
**120 min**	0.80 ± 0.00%	18.7 ± 0.9%	49.7 ± 1.8%	70.9 ± 0.6%	64.5 ± 0.5%
**180 min**	0.93 ± 0.23%	36.3 ± 1.2%	61.5 ± 0.6%	72.9 ± 0.6%	66.9 ± 0.5%
**24 h**	2.93 ± 0.23%	71.1 ± 0.2%	77.7 ± 0.9%	80.1 ± 0.2%	77.2 ± 0.4%
**48 h**	3.47 ± 0.46%	75.2 ± 0.0%	79.9 ± 0.8%	81.6 ± 0.0%	79.2 ± 0.0%
